# Kanamycin and G-Quadruplexes: An Exploration of Binding Interactions

**DOI:** 10.3390/molecules29245932

**Published:** 2024-12-16

**Authors:** Gianmarco Gualtieri, Emanuele Liborio Citriniti, Roberta Rocca, Valentina Arciuolo, Jussara Amato, Antonio Randazzo, Stefano Alcaro

**Affiliations:** 1 Dipartimento di Scienze della Salute, Università “Magna Græcia” di Catanzaro, Viale Europa, 88100 Catanzaro, Italy; g.gualtieri@unicz.it (G.G.); emanueleliborio.citriniti@unicz.it (E.L.C.); alcaro@unicz.it (S.A.); 2 Net4Science SRL, Università “Magna Græcia” di Catanzaro, Viale Europa, 88100 Catanzaro, Italy; 3 Department of Pharmacy, University of Naples Federico II, 80131 Naples, Italy; valentina.arciuolo@unina.it (V.A.); jussara.amato@unina.it (J.A.); antonio.randazzo@unina.it (A.R.)

**Keywords:** G-quadruplex, kanamycin, drug repurposing, docking, molecular dynamics

## Abstract

G-quadruplexes (G4s) are distinctive four-stranded nucleic acid structures formed by guanine-rich sequences, making them attractive targets for drug repurposing efforts. Modulating their stability and function holds promise for treating diseases like cancer. To identify potential drug candidates capable of interacting with these complex DNA formations, docking studies and molecular dynamics (MDs) simulations were conducted. Our analysis revealed kanamycin’s ability to bind to various G4 structures, offering valuable insights into its potential as a modulator of G4 activity. Kanamycin exhibited favorable interactions with both parallel and hybrid G4 topologies in human structures, suggesting a broader mechanism of action for aminoglycosides. These findings may also shed light on aminoglycoside-associated toxicities, indicating that their effects might extend to binding non-ribosomal RNA structures. In summary, this research highlights kanamycin’s potential as a promising tool for influencing G4 dynamics, paving the way for innovative therapeutic strategies targeting G4-related pathways.

## 1. Introduction

The involvement of G-quadruplex (G4) structures in key genomic functions, including transcription, replication, genome stability, and epigenetic regulation, as well as their numerous connections to cancer biology, is now well understood and extensively documented. These advancements have collectively driven research into G4 mechanisms, revealing new opportunities for therapeutic intervention [1,2]. Non-canonical base pairing in guanine-rich DNA and RNA sequences can give rise to G-quartets, stabilized by hydrogen bonds (H-bonds) involving “Watson–Crick” and “Hoogsteen” configurations. These G-quartets stack to form G4 structures (G4s), further stabilized by cations such as K^+^, Na^+^, and NH_4_^+^. The formation and stability of G4s are correlated to a combination of hydrogen bonding, metal coordination, and π–π stacking interactions, with their loop regions contributing to a wide range of structural diversity [1,3,4,5]. G4s interfere with essential cellular processes, including DNA replication, transcription, and recombination. They are frequently found in specific genomic regions, such as telomeres and oncogene promoters, while they are less prevalent in tumor suppressor genes. In vitro studies have explored the formation of intramolecular G4s in various human gene promoter regions, including those of the c-myc, c-kit, and bcl-2 genes, which play significant roles in cancer development [6,7,8]. As a result, targeting G4s within gene promoter regions presents a promising anticancer strategy with significant, yet largely unexplored, potential. The unique biophysical and structural characteristics of many gene promoter G4s make them ideal candidates for drug development, and their structural diversity offers the opportunity for highly selective therapeutic interventions [9,10]. Also, telomeric G4s play a crucial role in cancer by stabilizing telomeres and interfering with telomerase activity, which can lead to genomic instability and contribute to the unchecked cellular proliferation characteristic of cancer [11,12].

The diversity of existing G4s is most evident in the variations in the size and characteristics of their grooves and loops [2]. A prominent example is the guanine-rich nuclease hypersensitivity element III1 (NHE III1), which is situated upstream of the c-myc oncogene’s P1 promoter, that acts as a transcriptional repressor. c-myc is highly expressed in various cancers and plays a critical role in tumor progression [13]. Several models for the G4 structure have been proposed, with recent studies supporting a parallel-stranded quadruplex model. Oligomers containing the four 3′ guanine runs are sufficient for G4 formation, and this topology has been confirmed by NMR spectroscopy. Mutations in the MYC22-G14T/G23T sequence restrict the formation of loop isomers, offering insights for designing compounds to target c-myc expression [13]. Moreover, the c-kit oncogene is a key target for treating gastrointestinal cancers [14]. The c-kit proto-oncogene encodes a tyrosine kinase receptor involved in cell growth regulation, and mutations or overexpression can lead to oncogenesis [15]. Gleevec (imatinib) effectively inhibits c-kit kinase activity in gastrointestinal stromal tumors (GIST), but resistance due to mutations has emerged, prompting the development of new inhibitors. Alternatively, transcriptional regulation could offer another approach to c-kit inhibition [16]. The c-kit oncogene features two guanine-rich regions, known as c-kit1 and c-kit2 [17,18]. Phan et al. discovered a novel G4 in the c-kit1 region with a unique folding topology. This G4 is distinguished by an isolated guanine that participates in forming the G-tetrad core, despite the presence of four three-guanine tracts. This distinctive structure provides a valuable platform for designing targeted ligands [15]. On the other hand, extended NMR studies reveal that the c-kit2 promoter adopts two distinct all-parallel G4 conformations in slow exchange. In a 20 mM K^+^ solution, it forms a monomeric G4 (form-I), while in a 100 mM K^+^ solution, it forms a novel dimeric G4 (form-II). Form-II features an unprecedented all-parallel-stranded topology, in which each strand spans two G4s, each containing three layers of G-tetrads in a 3′ to 5′ orientation, with stacking continuity maintained by a non-canonical A•A pair, which forms a “sandwich” between the two G4s. This dimeric G4 may play a role in strand exchange during recombination events in guanine-rich regions [17]. While c-kit1 and c-kit2 have been extensively studied, there is limited information on c-kit*, which plays a crucial role in regulating c-kit transcription and contains consensus sites for the transcription factors SP1 and AP2. Kotar et al. used various spectroscopic and biophysical techniques to show that kit* adopts an antiparallel chair-type G4 with two G-quartets in physiologically relevant KCl concentrations. The presence of K^+^ ions stabilizes the structure through stacking interactions and specific base pairs, while Na^+^ and NH_4_^+^ ions also stabilize the pre-formed structure, aligning the kinetics of G4 formation with transcriptional processes [19]. Also, the Bcl-2 gene product, a mitochondrial membrane protein, is crucial for cell survival, inhibiting apoptosis and maintaining balance with other apoptosis-related proteins. Dysregulation of Bcl-2 is linked to various cancers, including lymphomas and solid tumors, and is associated with poor prognosis and treatment resistance [19]. Recent research by Dai et al. revealed that the BCL-2 promoter region forms a well-defined mixed parallel/antiparallel G4 structure with three G-tetrads, stabilized by specific loop conformations. This unique G4 structure suggests a role in gene regulation and potential as a target for drug development [20]. Among G4 structures, telomeric sequences stand out for their high structural polymorphism, allowing them to adopt various forms depending on sequence variations, environmental conditions, and cation presence [2]. The main topologies of telomeric G-quadruplexes include parallel, antiparallel, and mixed-strand arrangements, each with distinct folding patterns and stability profiles. Among these, the monomeric human telomere DNA quadruplex with a 3 + 1 strand fold topology is particularly abundant in potassium ion solutions, reflecting its stability and prevalence in physiological conditions [2].

In this context, several studies, including those conducted in our laboratory, have employed in silico screening to identify drug-like ligands targeting G4s. These investigations often reveal compounds with unique characteristics that diverge from traditional chromophore or side-chain structures [21,22,23,24,25,26,27,28,29]. Moreover, several drugs have also been assessed for their potential to interact with G4 structures, as these interactions can play a crucial role in their therapeutic efficacy. By evaluating drugs for their ability to bind to and stabilize these structures, researchers aim to uncover novel therapeutic mechanisms and enhance the specificity and effectiveness of treatments for various diseases [30,31,32,33,34].

Taking this into account, we conducted a Virtual Screening (VS) campaign of FDA-approved drug compounds against G4 structures found in various oncogene promoters and telomeres. The aim was to identify novel ligands that can interact with and potentially disrupt their oncogenic functions. Additionally, we aim to uncover new mechanisms of action for these drugs to better understand their therapeutic effects.

## 2. Results

### 2.1. Virtual Screening (VS)

To investigate the interactions between approved clinical drugs and the G4 structures found in certain oncogene promoters (Bcl-2, c-kit1, c-kit2, c-myc, and kit*) and the human telomeric sequence (mtel24), we performed a Structure-based Virtual Screening (SBVS). For each guanine-rich sequence, we identified the PDB model that matched the sequence used in later experimental tests. From these models, we selected the most stable conformations based on energy calculations for further analysis (Table S1). A total of 2568 FDA-approved drugs were screened against the best energy conformation of each G4 model by using the Glide Extra Precision (XP) protocol. The top 20 hits were selected for each model.

**Figure 1:**
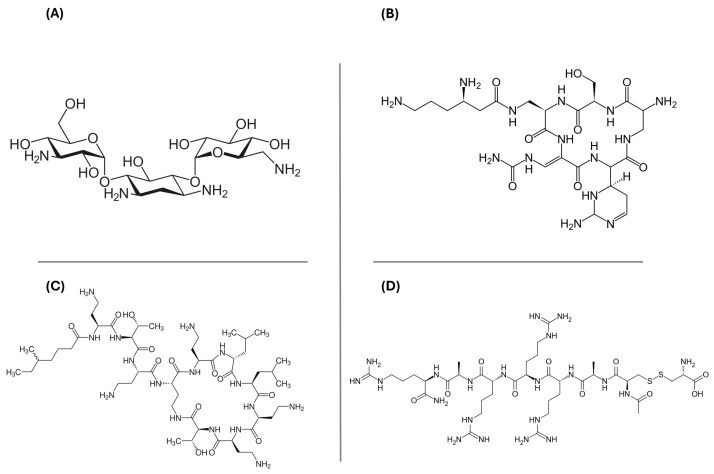
Two-dimensional chemical structures of the four hits obtained after the post-docking clustering step: (**A**) kanamycin, (**B**) capreomycin, (**C**) colistin, and (**D**) etelcalcetide.

For each model, the top 20 compounds were selected based on their XP G-score rankings (Table S2). The XP G-score values ranged from −11.06 to −15.71 kcal/mol for Bcl-2, −11.89 to −15.79 kcal/mol for c-kit1, −10.24 to −13.25 kcal/mol for c-kit2, −11.93 to −15.42 kcal/mol for c-myc, −10.01 to −14.06 kcal/mol for kit*, and −14.10 to −10.97 kcal/mol for htelo. Our analysis focused on the 12 drugs shared across all the G4 models under investigation. These compounds were clustered to identify the most significant lead structures from the VS, highlighting four predominant chemical scaffolds, as illustrated in Figure 1. Kanamycin, capreomycin, and colistin are antibiotic agents, while etelcalcetide is a calcium-sensing receptor agonist. Capreomycin belongs to the class of cyclic peptide antibiotics; it is used in the treatment of tuberculosis in combination with other drugs and it must be administered only parenterally. Colistin is a polymyxin antibiotic used to treat bacterial infections caused by susceptible Gram-negative bacteria. It is less toxic than polymyxin B, but overdosage can cause a neuromuscular blockade that may lead to apnea, respiratory arrest, and death. Etelcalcetide is a calcimimetic drug approved for the treatment of secondary hyperparathyroidism in adult patients who need hemodialysis.

Instead, kanamycin is commonly used to treat various infections caused by susceptible bacteria. It is available in oral, intramuscular, and intravenous forms. Kanamycin belongs to the class of aminoglycoside antibiotic agents, with a 2-deoxystreptamine core which can bind specifically to the bacterial 30S ribosomal subunit. Recently, Ranjan and Arya pointed out the crucial role played both by one of the protonated amines of the neomycin and the ring I in driving the interaction of aminoglycosides towards non-canonical DNA. These findings may give a possible explanation of some of the undesirable effects of aminoglycosides and promote the design of novel G4 binders with higher affinity [35].

### 2.2. Thermodynamic Analysis of Kanamycin–G4 Complexes

After a thorough evaluation of recent evidence, kanamycin was chosen for comprehensive thermodynamic analysis across all G4 models due to its comparatively low toxicity, improved compliance, and broader applicability compared to the other three scaffolds identified through clustering. We first assessed the theoretical kanamycin’s binding free energy (ΔG_bind_) across all G4 models. As shown in Table 1, the theoretical ΔG_bind_ values ranged from −43.81 to −64.71 kcal/mol. The mtel24–kanamycin complex demonstrated the most favorable thermodynamic score, while the c-kit2–kanamycin complex exhibited the least favorable binding energy among the G4 structures. Analysis of the individual contributions to the theoretical ΔG_bind_ revealed that coulombic interactions (ΔG_coul_) were the primary driving force behind kanamycin’s interaction with G4, with a peak value of −786.47 kcal/mol observed in the Bcl-2 promoter.

**Figure 2:**
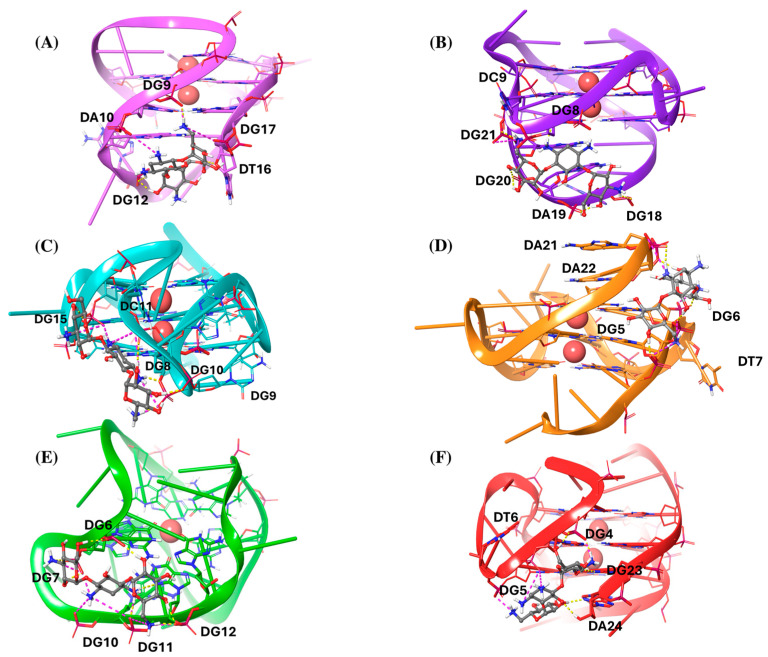
Three-dimensional (3D) representations of the most thermodynamically stable complexes of kanamycin with (**A**) Bcl-2, (**B**) c-kit1, (**C**) c-kit2, (**D**) c-myc, (**E**) kit*, and (**F**) mtel24 G4s. Kanamycin is depicted as a gray ball-and-sticks model. The nucleic acids are shown as faded plum, violet, cyan, orange, green, and red cartoons for Bcl-2, c-kit1, c-kit2, c-myc, kit*, and mtel24, respectively. All the residues are shown as sticks. K^+^ ions are represented as red spheres. Hydrogen bonds are indicated by dashed yellow lines, and salt bridges by dashed magenta lines.

Afterwards, we analyzed the interaction patterns of the most thermodynamically stable complexes, as shown in Figure 2.

Notably, kanamycin predominantly acts as a groove or loop binder across all G4 structures (Figure 2). The most frequent interactions observed are hydrogen bonds (H-bonds) and salt bridges, aligned with the most favorable ΔG_Coul_ values. In the Bcl-2–kanamycin complex, the ligand interacts with the major loop and partially with the groove II, by establishing three salt bridges with the phosphodiesterase portion of the residues DG9, DA10, and DG17, respectively. Additionally, it forms different H-bonds with the phosphodiesterase part of DG9 and DG12, as well as with the sugar portion of DT16 (Figure 2A). In the c-kit1–kanamycin complex, the ligand binds the five-residue stem–loop, by forming several H-bonds with the phosphate part of DG20, DA19, and DG18, and with the nucleobase of DG8. At the same time, it establishes two electrostatic interactions with the phosphate portion of DC9 and DG21 (Figure 2B). Regarding the c-kit2–kanamycin complex, the ligand widely interacts with the second loop, composed of the five nucleotide-segment DC9–DG10–DC11–DG12–DA13. In particular, it joins in multiple salt bridges with the phosphodiesterase portion of DC9, DC11, and DG15. Additionally, it engages in distinct H-bonds with the phosphate fragments of DG8, DG10, and DC11 (Figure 2C). On the other hand, in the complex with c-myc, kanamycin acts as a groove binder and also shows additional interaction with the two terminal residues, DA21 and DA22. In particular, it forms salt bridges and H-bonds with the phosphodiesterase part of DG5, DG6, DT7, DA21, and DA22 (Figure 2D). As regards the c-kit*–kanamycin complex, the ligand interacts with the groove formed by the residues DG6, DG7, DG11, and DG12, where it establishes salt bridges and H-bonds with the phosphate backbone. Additionally, kanamycin engages in electrostatic interactions with the phosphate groups of DG7 and DG10, located within a flexible loop region, further stabilizing the binding (Figure 2E). Finally, in the complex with mtel24, kanamycin forms electrostatic interactions with the phosphate part of DG4, DG5, and DG6. It is also involved in H-bonds with the phosphodiesterase portion of DG4, with the nucleobase fragment of DG23, and with both the nucleobase and sugar part of DA24 (Figure 2F).

### 2.3. Characterization of the Dynamic Interactions in G4s–Kanamycin Complexes

Drawing on insights from the literature and noting the lack of characteristic π–π interactions typically observed with G4 ligands, we performed 500 ns of molecular dynamics simulations (MDs) to explore kanamycin’s binding behavior across the different G4 structures investigated.

Firstly, we analyzed the RMSD trends for the entire structures of both DNA and kanamycin, focusing on their heavy atoms (Figure S1). This analysis was conducted by superimposing the G-core of each G4 structure. As shown in Figure S1A, kanamycin induced a significant conformational change in the c-kit2 G4 structure, which achieved geometrical convergence after 150 ns. Similarly, the c-myc structure exhibited a conformational shift after 200 ns, which remained stable throughout the rest of the simulation. The other G4 structures followed a comparable RMSD trend, with values ranging between 1 to 4 Å. Kanamycin’s RMSD trend not only reflects its high flexibility, but also underscores its ability to adapt and bind to G4 structures across different conformations and binding modes (Figure S1B). Among all the kanamycin complexes, the mtel24 G4 structure showed a complete loss of interactions with the ligand, indicating a significantly lower affinity for this target compared to the other G4 structures. A lot of fluctuations throughout the MDSs were also observed when kanamycin binds the c-ki1 G4 structure, indicating a lower stability for this complex. Conversely, c-kit*, Bcl-2, c-kit2, and c-myc showed a similar RMSD behavior, with a high change in the first part of the simulation, that could indicate the necessity for the ligand to overcome an induce-fit process when binding these structures. After 100 ns, kanamycin achieves a geometrical convergence. Notably, both the c-kit2 and c-myc G4 structures exhibited RMSD trends similar to those of the other DNA promoters, with significant fluctuations in the early stages of the MDs simulations, followed by stabilization as the simulations progressed.

**Figure 3:**
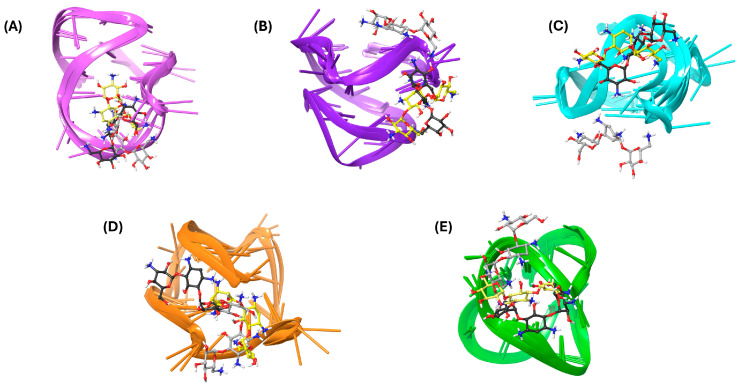
Three-dimensional (3D) representations of the most populated MDS clusters of kanamycin in complex with (**A**) Bcl-2, (**B**) c-kit1, (**C**) c-kit2, (**D**) c-myc, and (**E**) kit*. The nucleic acids are represented as faded plum, violet, cyan, orange, and green cartoons corresponding to Bcl-2, c-kit1, c-kit2, c-myc, and kit*, respectively. Kanamycin is illustrated as a ball-and-stick model, with yellow, black, and gray colors denoting cluster 1, cluster 2, and cluster 3, respectively.

Finally, a clustering analysis was performed to identify the most common kanamycin conformations and orientations within the G4 structures observed during the MDSs (Figure 3), excluding mtel24 due to disrupted interactions. The simulations characterized kanamycin as a binder to loops or grooves. For the Bcl-2 G4 structure, the second loop was confirmed as the primary binding site for kanamycin (Figure 3A), with the key interacting residues DG12, DA13, and DA14. In the c-kit1 structure, clusters 1 and 2 occupied the groove formed by DG6 and DG7, while also interacting with DG18 from the third loop. Conversely, cluster 3 shifted to the upper position, engaging with DG10, DC11, and DA5 (Figure 3B). In the c-kit2 simulations, the two most representative clusters revealed a binding site in the groove formed by DG14 and DG15, establishing hydrogen bonds with DT12 and DA13. In contrast, cluster 3 exhibited a shifted binding site toward the bottom, interacting with DC9, DG10, and DG16 (Figure 3C). For c-myc, kanamycin was frequently positioned between the second loop and the 3′-terminal, forming multiple interactions with DT7, DG9, DG10, and DA22 (Figure 3D). Finally, the most representative structure of the c-kit*–kanamycin complex revealed a binding site near the second loop, with the ligand forming numerous favorable contacts with DG7, DG9, DG10, and DG11 (Figure 3E).

### 2.4. In Vitro Study of Interactions Between Kanamycin and G4s

To assess the binding of kanamycin to selected G4s, 1D ^1^H-NMR experiments were performed. The spectral regions corresponding to the imino and aromatic protons of the G4 structures in the absence and presence of kanamycin are shown in Figure S2. According to the literature, under the experimental conditions used, Bcl-2, c-kit1, c-kit2, c-myc, and mtel24 adopt a single G4 conformation characterized by 12 well-resolved imino proton peaks, indicative of the 12 guanines involved in the three G-tetrad planes [13,17,20,36,37]. Conversely, c-kit* showed eight well-resolved imino proton peaks, consistent with a G4 structure comprising two G-tetrad layers [19]. In general, little or no changes were observed in the imino proton signals, indicating that kanamycin does not interact with the G-tetrads, consistent with its inability to participate in stacking interactions. However, slight localized variations in specific proton signals may suggest interactions of kanamycin with bases located in the loops or grooves of G4 structures. In particular, kanamycin binding to Bcl-2 G4 affected the aromatic proton signals of the residues G11, G12, A13, and A14, suggesting interaction with the second loop of this G4. For c-kit1 G4, small changes were observed in the imino protons of G10, G13, G14, and G15, along with G22, suggesting kanamycin binding to this region. Changes of some aromatic proton signals were also noted, but the lack of signal assignment prevented detailed attribution. For c-kit2 G4, slight variations were detected in both the imino and aromatic protons of G14, as well as the aromatic protons of A13 and T12, suggesting that kanamycin may interact with this side of G4. On the other hand, the addition of kanamycin to c-myc G4 caused slight changes in both the imino and aromatic protons of the guanines G16, G17, and G18, along with the aromatic proton of T19, suggesting an interaction in this region. For mtel24, no significant changes were detected in the imino or aromatic proton regions after the addition of kanamycin, apart from subtle shifts in the aromatic protons of G11 and T12, suggesting the involvement of the second loop. Finally, for c-kit*, the disappearance of the peak at 13.5 ppm corresponding to the base pair between G10 and C18 (located in the second loop and in the 3′ flanking region of the G4, respectively), together with slight shifts in the imino proton peaks of G16 and G17, suggest the binding of kanamycin in this region. Overall, the small and localized variations observed suggest weak and limited interactions between kanamycin and G4s that preclude accurate quantification of these interactions.

## 3. Discussion

The literature contains numerous studies on drug repurposing [38,39,40], including some focused on G4s. However, most of these studies have primarily concentrated on the G4 associated with the COVID-19 pandemic [32,41,42].

In this study, we conducted an in silico investigation of kanamycin binding to various physiological G4 structures, based on results from a VS of the Drug Bank database. In the literature, ligands that interact with G4 structures are typically distinct from kanamycin, as they often feature an extended aromatic region. This is vital for forming π–π interactions, which are generally essential for stabilizing the complex [43,44]. Conversely, the binding of kanamycin is primarily driven by electrostatic interactions, likely resulting from the protonation of one of its amine groups. Kanamycin appears to function as a binder to loops or grooves, establishing several electrostatic interactions, including salt bridges and hydrogen bonds. MDSs of 500 ns were influenced by the flexible behavior of kanamycin. However, they revealed a more stable complex with c-kit*, Bcl-2, c-kit2, and c-myc, while significant fluctuations were observed in the complex with c-kit1. Although the thermodynamic analysis indicated that the complex with mtel24 was the most favorable, it proved unstable during the simulations, leading to kanamycin detaching from the G4 structure and breaking all interactions. Recently, Rajan et al. reported that another aminoglycoside, neomycin, can bind not only to various RNA structures but also to higher-order non-canonical DNA structures such as G4s. They specifically investigated the interactions of several aminoglycosides, including kanamycin, with a parallel G4 derived from a Tetrahymena telomere [35]. In our VS, neomycin did not rank among the top 20 hits, whereas kanamycin emerged as the most promising aminoglycoside after clustering analysis. We explored kanamycin’s binding interactions with different human G4 structures, yielding favorable results for both parallel and hybrid topologies. The binding of kanamycin to selected G4s was further examined in vitro using one-dimensional proton NMR spectroscopy, which confirmed that kanamycin interacts with the loops or grooves of these structures. These findings may offer alternative explanations for the toxicities associated with aminoglycosides, suggesting their potential binding to non-canonical DNA structures like G4s. Moreover, they could pave the way for new therapeutic strategies targeting G4s using kanamycin and its derivatives.

In conclusion, these results highlight kanamycin’s potential as a promising agent for influencing G4 dynamics, offering new possibilities for developing innovative therapeutic approaches aimed at targeting G4-associated pathways.

## 4. Materials and Methods

### 4.1. Database Preparation

To identify commercially available drugs that stabilize DNA G4s, we selected 2568 Food and Drug Administration (FDA)-approved drugs for human use from the Drug Bank database [45]. All the compounds were prepared using the LigPrep platform [46]. Hydrogens were added, salts were removed, and ionization and tautomerization states were calculated using Epik at pH 7.4. Subsequently, an energy minimization was carried out on the 3554 three-dimensional structures using the OPLS3 force field [47].

### 4.2. Receptor Preparation and NMR Conformer Selection

The nuclear magnetic resonance (NMR) solution structures of G4s formed in the promoter regions of Bcl-2, c-kit1, c-kit2, c-myc, and kit*, as well as the human telomeric sequence (htelo), were obtained from the Protein Data Bank [48] using the following accession codes: 2F8U [20], 2O3M [15], 2KYP [36], 1XAV [13], 6GH0 [19], and 2GKU [37], respectively. The structure 2F8U consists of 10 conformers representing a well-defined mixed parallel/antiparallel-stranded G4 structure in the human Bcl-2 promoter region [20]. 2O3M contains 11 conformers of a monomeric intramolecular G4 formed by a G-rich sequence in the c-kit promoter [15]. 2KYP includes 12 conformers of a monomeric G4 from the human c-kit2 proto-oncogene promoter [36]. 1XAV is made up of 20 conformers of a monomeric parallel-stranded G4 found in the human c-myc promoter [13]. 6GH0 consists of 10 conformers representing an antiparallel G4 with two G-quartets located in the kit* promoter region [19]. Lastly, 2GKU is composed of 12 conformers of a monomeric human telomeric DNA G4 with a 3 + 1 strand fold topology [37]. All the aforementioned experimental structures were selected because they perfectly match the sequences used in subsequent biophysical tests designed to verify ligand binding. This ensures consistency between the structural analysis and the ligand-binding studies of G4 formations in various promoter regions and the human telomeric sequence. Thus, they were prepared and refined by means of the Protein Preparation Wizard tool [49] implemented in Maestro using OPLS3 as the force field [47]. The correct bond orders were assigned, and all the hydrogen atoms were added.

To select the most suitable conformer for molecular modeling studies, all conformers extracted directly from the PDB entries of each G4 NMR PDB model were evaluated through energy calculations using MacroModel v 7.8 [50], without performing any structural optimization. The Current Energy panel was employed to determine the molecular mechanics energy of each structure in the solution phase, utilizing the OPLS3 force field with water as the solvation model. The conformers were ranked based on their Total Energy (kcal/mol), and only the most stable conformers were retained for further molecular modeling analysis (Table S1).

### 4.3. Docking Protocol

The most stable conformer for each PDB model was then used to generate the grids for the molecular docking studies. Each energy grid was built centering the docking box on the G-tetrads’ centroid and setting its outer box size to 40 × 40 × 40 Å. For each docking run, 10 poses per ligand were generated and the scaling factor for the target Van der Waals radii was set to 0.8. We used the Extra Precision (XP) scoring function of the Glide ver. 7.8 software of the Schrödinger suite [51] to screen the commercially available drugs database against G4 promoters. Then, the top 20 *hits* from each docking run were selected based on their XP Glide scores. From these, we focused on the drugs that demonstrated effective interactions with all the G4 promoters and the telomeric sequence under investigation. To refine the selection and choose a few drugs for further investigation, we used the Canvas Similarity and Clustering Tool in Maestro to group the *hits* based on similarity metrics [52]. Hierarchical agglomerative clustering was applied using the ‘Linear’ fingerprint type and the ‘Centroid’ linkage method, with the number of clusters set to 4. The program was instructed to output the structures nearest to the centroid of each cluster.

Subsequently, all the complexes between the G4 targets and the most promising *hit* obtained after clusterization were further submitted to the Molecular Mechanics Generalized Born/Surface Area (MM-GBSA) method [53], applying molecular mechanics and continuum solvation models, to compute their binding free energy (ΔG_bind_).

### 4.4. Molecular Dynamics Simulation (MDS) Protocol

For each G4 structure, the best thermodynamics complex with kanamycin was submitted to 500 ns of MDs simulation using the GROMACS ver. 5.1.4 package [53]. The G4 structures were modeled using the *Parm99* Amber force field with the *parmbsc0* refinement for nucleic acids [54]. For the ligand, the electrostatic potential (ESP) was first calculated using Jaguar version 9.3 [55] with the 6-31G* basis set at the Hartree–Fock level of theory. The restrained electrostatic potential (RESP) [56] charges were then determined with Antechamber [57], and the ligand was parameterized using the General Amber Force Field (GAFF) [58]. The systems were placed in a cubic box with a buffering distance between the edges of the box and the DNA of 10 Å. The TIP3P water model was chosen as the explicit solvent model. K^+^ counterions were added to the system until charge neutralization. The long-range electrostatic interactions were computed by means of the Particle Mesh Ewald (PME) method [59], using a 1.25 Å grid spacing in periodic boundary conditions. The so prepared systems were then submitted to a double minimization step: (i) minimization of water and ions applying position restraints on the complex; and (ii) global minimization without any position restraints. Thus, all systems were initially equilibrated for 5 ns in the NVT ensemble, followed by equilibration in the NPT ensemble at 300 K and 1 atm. Subsequently, 500 ns of MDS production run in the NPT ensemble was carried out with V-rescale as the thermostat and Parrinello–Rahman as the barostat. An integration time step of 2 fs was used. Trajectory frames were collected every 20 ps and were analyzed using the GROMACS tools. For each trajectory, the clustering process was performed using cpptraj from AmberTools 12 [60], analyzing all frames from each molecular dynamics (MDs) simulation, which amounted to 25,000 structures. The clustering algorithm employed was hierarchical agglomerative (bottom-up), and the process was terminated when the minimum distance between clusters exceeded 3 Å or when only 10 clusters remained. Average linkage, which computes the average distance between members of two clusters, was used as the clustering method, with the RMSD of kanamycin serving as the distance metric.

### 4.5. 1D ^1^H-NMR Experiments

The following DNA sequences were used in this study:

5′-GGGCGCGGGAGGAATTGGGCGGG-3′ (Bcl-2); 

5′-AGGGAGGGCGCTGGGAGGAGGG-3′ (c-kit1);

5′-CGGGCGGGCGCTAGGGAGGGT-3′ (c-kit2);

5′-GGCGAGGAGGGGCGTGGCCGGC-3′ (c-kit*);

5′-TGAGGGTGGGTAGGGTGGGTAA-3′ (c-myc); 

5′-TTGGGTTAGGGTTAGGGTTAGGGA-3′ (mtel24). 

Oligonucleotide sequences were chemically synthesized and purified as previously described [61,62,63]. DNA samples for nuclear magnetic resonance (NMR) spectroscopy were prepared at a 20–50 μM final oligonucleotide concentration, in 250 μL (H_2_O/D_2_O 9:1) buffer solution (5 mM potassium phosphate buffer (pH 7.0) containing 20 mM KCl). To ensure proper folding, oligonucleotides were annealed by heating at 95 °C for 5 min, followed by slow cooling to room temperature and overnight storage at 4 °C. DNA/kanamycin mixtures were obtained by adding increasing amount of kanamycin (ranging from 0.5 to 2 molar equiv. in respect to G4) directly to the DNA solution inside the NMR tube [64], followed by an equilibration of 15 min before acquiring the spectra. NMR experiments were performed on a Bruker Advance NEO NMR spectrometer (Bruker BioSpin, Rheinstetten, Germany) operating at 600 MHz (^1^H Larmor frequency), equipped with a cryo-probe set and a cooled SampleJet autosampler. Data were processed using the vendor software TOPSPIN 4.0.7 (Bruker Biospin Gmbh, Rheinstetten, Germany).

## Figures and Tables

**Table 1:** Theoretical ΔG_bind_ and the related single contributions of the binding free energy of the most thermodynamically favorable complex of kanamycin with all G4 models. All thermodynamic values are reported in kcal/mol.

G4 Model	ΔG_bind_ (kcal/mol)	ΔG_coul_ (kcal/mol)	ΔG_lipo_ (kcal/mol)	ΔG_vdw_ (kcal/mol)	ΔG_solv_ (kcal/mol)
Bcl-2	−58.89	−786.47	−7.86	−29.44	759.36
c-kit1	−53.94	−770.08	−7.82	−23.74	740.36
c-kit2	−43.81	−734.77	−5.75	−13.77	696.23
c-myc	−48.87	−663.10	−8.58	−22.07	632.21
kit*	−53.84	−754.97	−7.90	−23.02	722.76
mtel24	−64.71	−752.54	−7.86	−24.36	712.58

## Data Availability

Data are contained within the article and Supplementary Materials.

## Supplementary Materials

The following supporting information can be downloaded at https://www.mdpi.com/article/10.3390/molecules29245932/s1, Table S1. Energetic values of the best NMR conformers for each G4 sequence used in this study, along with their corresponding PDB codes; Table S2. Drug Bank Code, generic name, and XP G-score of the top 20 compounds selected for Bcl-2 G4 after docking simulations; Table S3. Drug Bank Code, generic name, and XP G-score of the top 20 compounds selected for c-kit1 G4 after docking simulations; Table S4. Drug Bank Code, generic name, and XP G-score of the top 20 compounds selected for c-kit2 G4 after docking simulations; Table S5. Drug Bank Code, generic name, and XP G-score of the top 20 compounds selected for c-myc G4 after docking simulations; Table S6. Drug Bank Code, generic name, and XP G-score of the top 20 compounds selected for c-kit* G4 after docking simulations; Table S7. Drug Bank Code, generic name, and XP G-score of the top 20 compounds selected for mtel24 G4 after docking simulations; Figure S1. (A) RMSD trends of the G4 structures induced by kanamycin. (B) RMSD trends of kanamycin, calculated using the ligand heavy atoms during MDSs and superimposing the G-core structures. RMSD values are reported in Å. Figure S2. Imino and aromatic proton regions of the 1D ^1^H NMR spectra of Bcl2, c-kit1, c-kit2, c-myc, mtel24, and c-kit* G4s titrated with kanamycin.
