# Supplementary material for: Kanamycin and G-Quadruplexes: An Exploration of Binding Interactions
Source: Molecules. 2024 Dec 16;29(24):5932. doi: 10.3390/molecules29245932 (PMC11676551; doi:10.3390/molecules29245932)
Supplement: Supplementary file 1 [file molecules-29-05932-s001.zip › molecules-3337756-supplementary.pdf]

Kanamycin and G-Quadruplexes: An Exploration of Binding Interactions

Gianmarco Gualtieri [1, ‡] *Emanuele Liborio Citriniti* [1, ‡] *Roberta Rocca* [1,2,*] *Valentina Arciuolo*[3] *Jussara Amato* [3], *Antonio Randazzo*[3] *and Stefano Alcaro* [1,2]

[1] Dipartimento di Scienze della Salute, Università “Magna Græcia” di Catanzaro, Viale Europa, 88100 Catanzaro, Italy;

[2] Net4Science SRL, Università "Magna Græcia" di Catanzaro, Viale Europa, 88100 Catanzaro, Italy;

[3] Department of Pharmacy, University of Naples Federico II, Naples, Italy.

Table S1. Energetic values of the best NMR conformers for each G4 sequence used in this study, along with their corresponding PDB codes.

G4	PDB ID	Total Energy (*kcal/mol*)
Bcl-2	2F8U	-3930.07
c-kit1	2O3M	-4031.23
c-kit2	2KYP	-4102.42
c-myc	1XAV	-4090.63
kit*	6GH0	-4369.55
mtel24	2GKU	-4271.36

Table S2. Drug Bank Code, generic name and XP G-score of the top 20 compounds selected for Bcl-2 G4 after docking simulations.

Bcl-2		
**Drug Bank Code**	**Generic Name**	**XP G-score (*kcal/mol*)**
DB00781	Polymyxin B	-15.71
DB00684	Tobramycin	-15.07
DB00452	Framycetin	-14.92
DB00479	Amikacin	-14.67
DB13274	Micronomicin	-14.26
DB11512	Dihydrostreptomycin	-14.03
DB13270	Dibekacin	-13.74
DB00955	Netilmicin	-13.71
DB01172	Kanamycin	-13.66
DB00314	Capreomycin	-13.53
DB00803	Colistin	-13.52
DB12615	Plazomicin	-13.43
DB00878	Chlorhexidine	-13.31
DB01421	Paromomycin	-12.84
DB03615	Ribostamycin	-12.31
DB00644	Gonadorelin	-11.80
DB01301	Rolitetracycline	-11.33
DB06809	Plerixafor	-11.12
DB12865	Etelcalcetide	-11.11
DB00738	Pentamidine	-11.06

Table S3. Drug Bank Code, generic name and XP G-score of the top 20 compounds selected for C-kit1 G4 after docking simulations.

c-kit1		
**Drug Bank Code**	**Generic Name**	**XP G-score (*kcal/mol*)**
DB00781	Polymyxin B	-15.79
DB00452	Framycetin	-15.27
DB00314	Capreomycin	-14.42
DB00803	Colistin	-14.32
DB13270	Dibekacin	-14.21
DB00684	Tobramycin	-14.15
DB13274	Micronomicin	-13.99
DB00644	Gonadorelin	-13.90
DB11700	Setmelanotide	-13.40
DB12615	Plazomicin	-13.33
DB00955	Netilmicin	-13.29
DB00035	Desmopressin	-13.17
DB12865	Etelcalcetide	-13.15
DB00878	Chlorhexidine	-13.12
DB03808	Hexamidine	-13.00
DB01172	Kanamycin	-12.48
DB01204	Mitoxantrone	-12.15
DB01082	Streptomycin	-12.07
DB00738	Pentamidine	-11.98
DB00479	Amikacin	-11.89

Table S4. Drug Bank Code, generic name and XP G-score of the top 20 compounds selected for C-kit2 G4 after docking simulations.

c-kit2		
**Drug Bank Code**	**Generic Name**	**XP G-score (*kcal/mol*)**
DB00803	Colistin	-13.25
DB00781	Polymyxin B	-13.25
DB00314	Capreomycin	-12.81
DB13270	Dibekacin	-12.40
DB13274	Micronomicin	-12.21
DB00684	Tobramycin	-12.12
DB12615	Plazomicin	-12.09
DB00452	Framycetin	-11.95
DB00479	Amikacin	-11.95
DB00955	Netilmicin	-11.86
DB12865	Etelcalcetide	-11.83
DB00290	Bleomycin	-11.76
DB01421	Paromomycin	-11.59
DB00284	Acarbose	-11.11
DB01082	Streptomycin	-10.97
DB00878	Chlorhexidine	-10.94
DB03615	Ribostamycin	-10.87
DB09487	Iotrolan	-10.82
DB01172	Kanamycin	-10.71
DB11700	Setmelanotide	-10.24

Table S5. Drug Bank Code, generic name and XP G-score of the top 20 compounds selected for C-myc G4 after docking simulations.

c-myc		
**Drug Bank Code**	**Generic Name**	**XP G-score (*kcal/mol*)**
DB12615	Plazomicin	-15.42
DB00684	Tobramycin	-14.60
DB02638	Terlipressin	-14.21
DB00781	Polymyxin B	-14.19
DB00803	Colistin	-14.11
DB13270	Dibekacin	-13.95
DB00479	Amikacin	-13.86
DB00644	Gonadorelin	-13.76
DB01172	Kanamycin	-13.92
DB00314	Capreomycin	-13.71
DB01421	Paromomycin	-13.30
DB13274	Micronomicin	-13.23
DB00452	Framycetin	-12.94
DB11761	Tenapanor	-12.84
DB00955	Netilmicin	-12.58
DB00290	Bleomycin	-12.48
DB12865	Etelcalcetide	-12.39
DB06663	Pasireotide	-12.20
DB03808	Hexamidine	-12.19
DB06791	Lanreotide	-11.93

Table S6. Drug Bank Code, generic name and XP G-score of the top 20 compounds selected for kit* G4 after docking simulations.

kit*		
**Drug Bank Code**	**Generic Name**	**XP G-score (*kcal/mol*)**
DB00781	Polymyxin B	-14.06
DB00803	Colistin	-14.04
DB00684	Tobramycin	-13.42
DB01172	Kanamycin	-13.37
DB00314	Capreomycin	-13.29
DB01082	Streptomycin	-13.12
DB13274	Micronomicin	-12.86
DB00479	Amikacin	-12.84
DB13270	Dibekacin	-12.78
DB12615	Plazomicin	-12.70
DB00452	Framycetin	-12.34
DB00290	Bleomycin	-12.23
DB00955	Netilmicin	-11.94
DB01421	Paromomycin	-11.77
DB11512	Dihydrostreptomycin	-11.64
DB00644	Gonadorelin	-11.53
DB12865	Etelcalcetide	-11.40
DB00035	Desmopressin	-10.68
DB03615	Ribostamycin	-10.66
DB15494	Edotreotide gallium Ga-68	-10.01

Table S7. Drug Bank Code, generic name and XP G-score of the top 20 compounds selected for mtel24 G4 after docking simulations.

**mtel24**		
**Drug Bank Code**	**Generic Name**	**XP G-score (*kcal/mol*)**
DB00803	Colistin	-14.10
DB00479	Amikacin	-13.96
DB00452	Framycetin	-13.90
DB01172	Kanamycin	-13.52
DB11512	Dihydrostreptomycin	-13.41
DB03615	Ribostamycin	-13.21
DB13270	Dibekacin	-13.09
DB00684	Tobramycin	-12.94
DB01421	Paromomycin	-12.81
DB00781	Polymyxin B	-12.62
DB00878	Chlorhexidine	-12.48
DB00314	Capreomycin	-12.48
DB12865	Etelcalcetide	-12.44
DB13274	Micronomicin	-12.37
DB02638	Terlipressin	-11.58
DB12615	Plazomicin	-11.56
DB06825	Triptorelin	-11.23
DB00955	Netilmicin	-11.20
DB09050	Ceftolozane	-11.11
DB00290	Bleomycin	-10.97

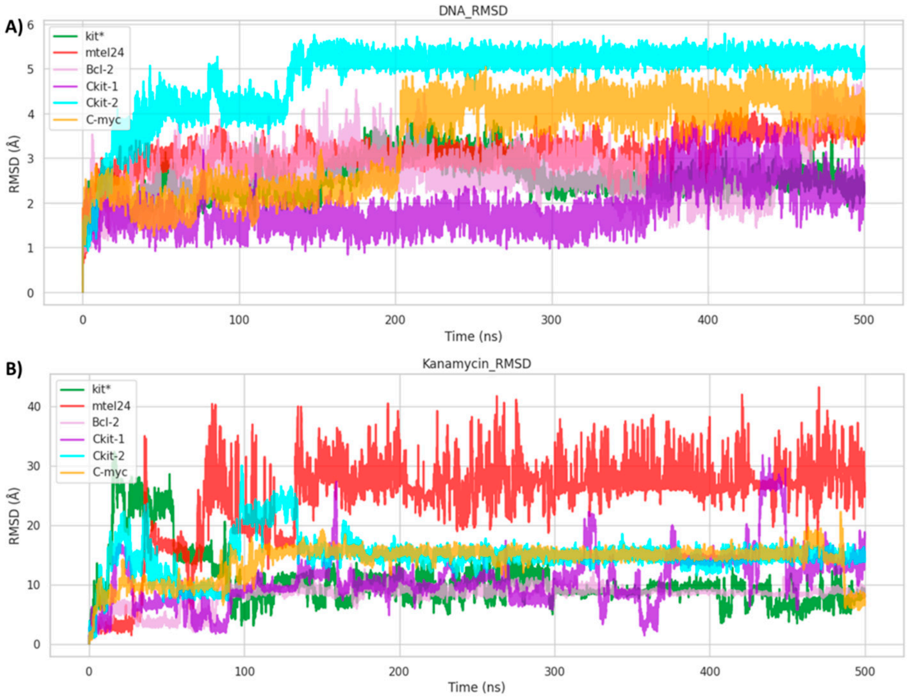

Figure S1. A) RMSD trends of the entire G4 structures induced by kanamicyn. B) RMSD trends of kanamicyn, calculated using the ligand heavy atoms during MDs and superimposing the G-core structures. RMSD values are reported in Å.

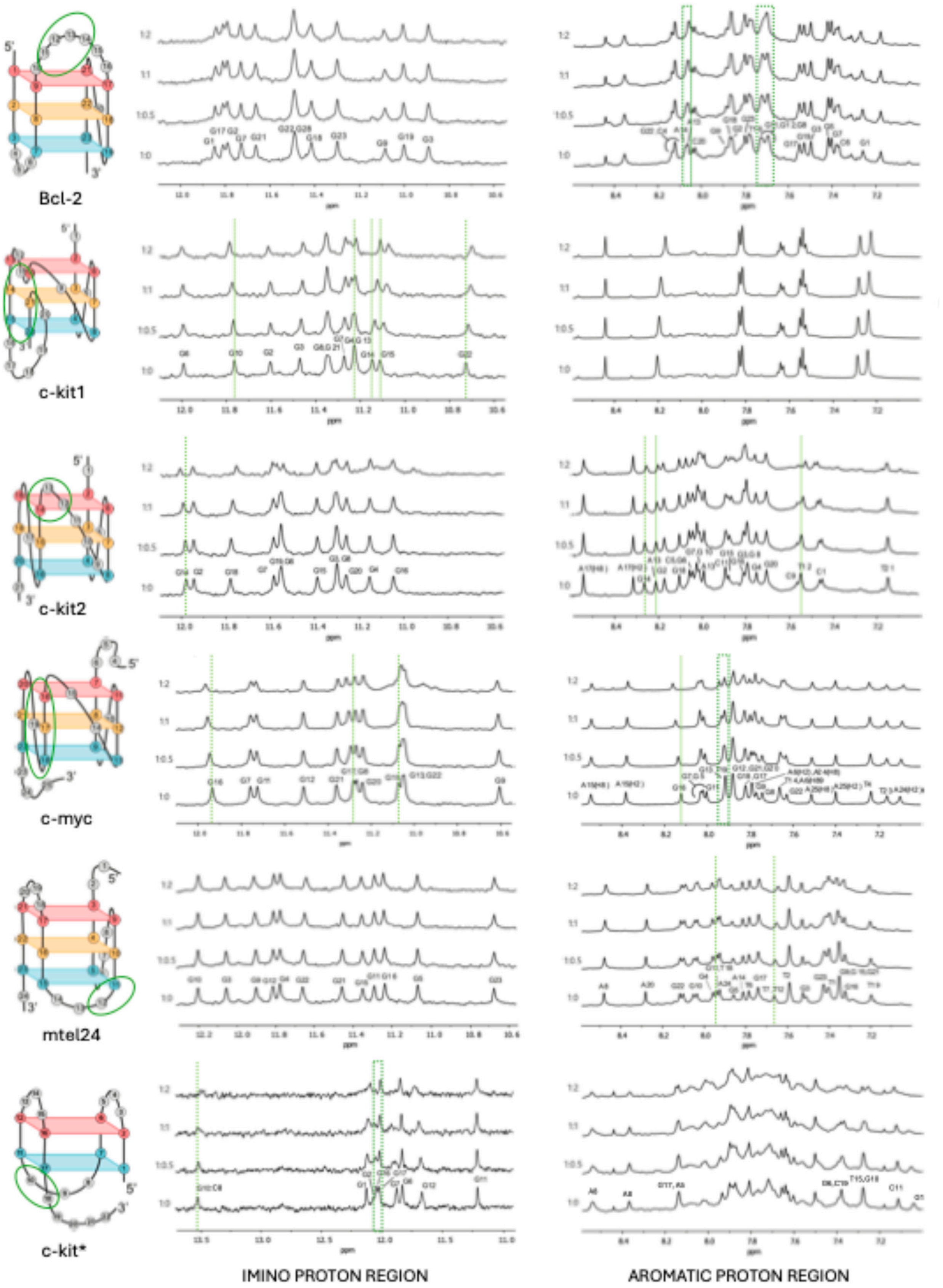

Figure S2. Imino and aromatic proton regions of the 1D ^{1}H NMR spectra of Bcl2, c-kit1, c-kit2, c-myc, mtel24, and c-kit* G4s titrated with kanamycin. The drug equivalents are shown on the left of each panel. Experiments were recorded at 25 °C in 5 mM KH_2PO_4/K_2HPO_4 buffer (pH 7.0) containing 20 mM KCl.
